# Smoking Cessation Inertia in Diabetes Care

**DOI:** 10.1111/1753-0407.70215

**Published:** 2026-04-05

**Authors:** Yusuff Adebayo Adebisi, Anoop Misra, Riccardo Polosa

**Affiliations:** ^1^ Department of Clinical and Experimental Medicine University of Catania Catania Italy; ^2^ College of Social Sciences University of Glasgow Glasgow UK; ^3^ Nuffield Department of Population Health University of Oxford Oxford UK; ^4^ Diabetes Foundation (India) New Delhi India; ^5^ National Diabetes, Obesity and Cholesterol Foundation (N‐DOC) New Delhi India; ^6^ Fortis C‐DOC Centre for Excellence for Diabetes, Metabolic Disease, and Endocrinology New Delhi India; ^7^ Center of Excellence for the Acceleration of HArm Reduction (CoEHAR) University of Catania Catania Italy; ^8^ Centre for the Prevention and Treatment of Tobacco Addiction (CPCT), University Teaching Hospital “Policlinico‐S. Marco” University of Catania Catania Italy

**Keywords:** cardiovascular risk, diabetes care integration, harm reduction, nicotine dependence, smoking cessation, tobacco use, type 2 diabetes

## 1 The Converging Epidemics of Diabetes and Tobacco

Type 2 diabetes (T2D) is one of the most pressing noncommunicable disease challenges globally. In 2022, an estimated 828 million adults were living with diabetes, an increase of approximately 630 million since 1990 [1]. This rapid rise has unfolded alongside another long‐standing epidemic: combustible tobacco use. According to the World Health Organization, approximately 1.3 billion people use tobacco worldwide, and tobacco causes more than 7 million deaths each year, most of them in low‐ and middle‐income countries.

The relationship between tobacco and T2D extends beyond simple co‐occurrence. Active smoking is an independent risk factor for incident T2D. Meta‐analytic evidence indicates a 37% higher risk among current smokers compared with never smokers, with a clear dose‐response gradient [2]. Unlike type 1 diabetes, which is autoimmune in origin, T2D has a well‐established causal link with smoking. The tobacco–T2D nexus therefore represents a preventable and modifiable driver of disease burden.

Modern diabetes care is increasingly structured and metrics‐driven. Clinicians routinely monitor glycemia, blood pressure, and lipid levels, stratify cardiovascular risk, and intensify treatment when targets are not achieved [3]. Yet smoking cessation, despite its well‐established role in reducing vascular injury and end‐organ damage, remains inconsistently integrated into routine diabetes care. In many settings, smoking status is recorded irregularly, addressed briefly, or deferred to follow‐up that never occurs [4]. As a result, a major modifiable exposure persists in a population already at elevated risk of complications. While diabetes care has successfully operationalized pharmacological and behavioral risk reduction across multiple domains, tobacco treatment remains marginal, fragmented, and weakly institutionalized.

## 2 Scale and Distribution of Tobacco Use in T2D

**FIGURE 1:**
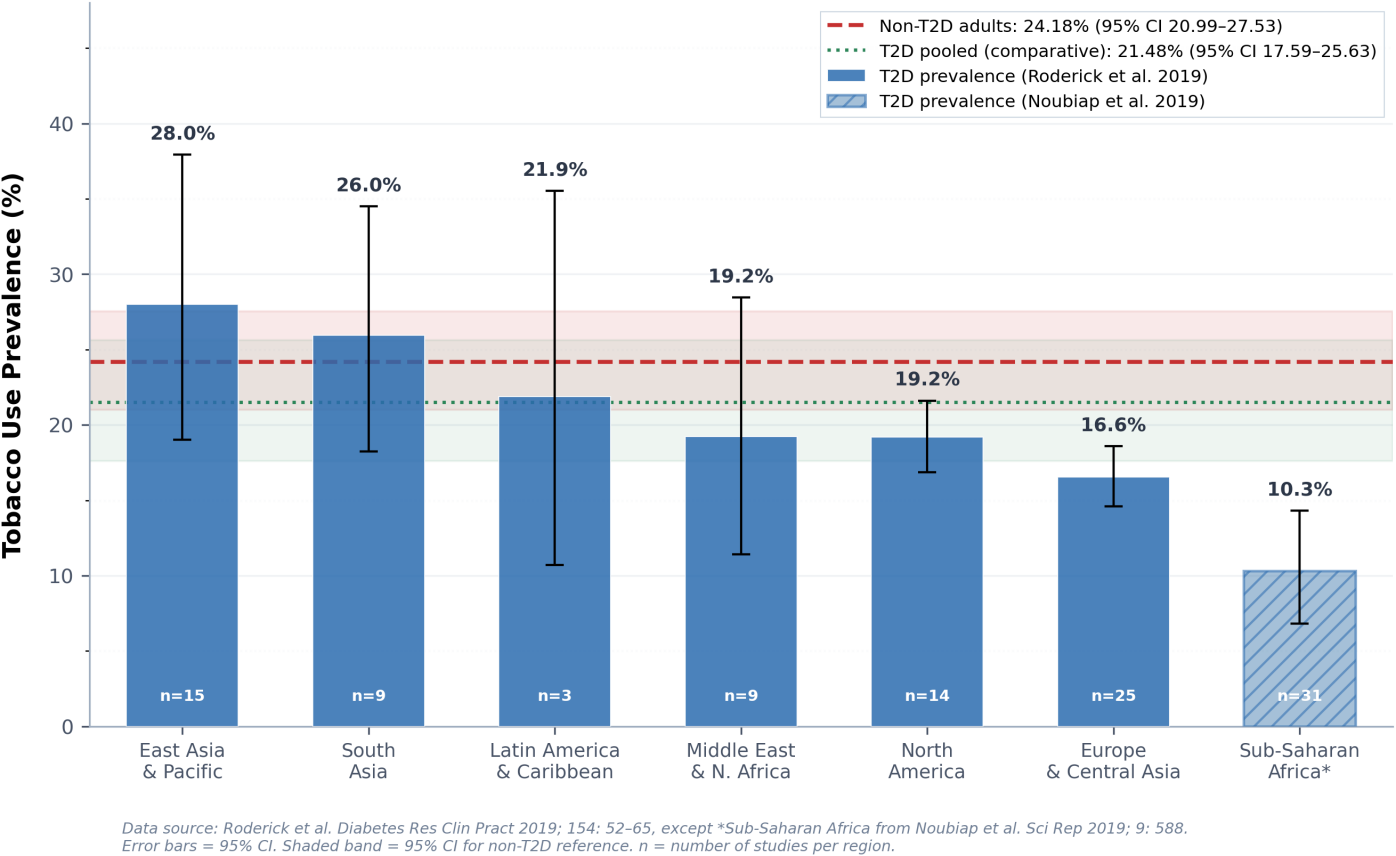
Tobacco Use Prevalence Among Adults With T2D by World Bank Region Compared With Non‐T2D Adults.

Tobacco smoking remains common among adults living with T2D worldwide. A systematic review and meta‐analysis spanning 74 studies across 33 countries, including approximately 3.2 million participants, estimated a global mean tobacco use prevalence of 20.8% among people with T2D [5]. Prevalence varied substantially by region (Figure 1). Estimates were highest in East Asia and the Pacific (28.0%), followed by South Asia (26.0%), Latin America and the Caribbean (21.9%), the Middle East and North Africa (19.2%), and North America (19.2%). Europe and Central Asia reported a lower prevalence of 16.6% [5]. A separate meta‐analysis from Africa estimated an overall active smoking prevalence among people with diabetes of 12.9%, with notable subregional variation: 21.3% in North Africa compared with 10.3% in Sub‐Saharan Africa [4].

Importantly, these estimates are broadly comparable to those observed in adults without diabetes. In the 33 studies within the same meta‐analysis that included a non‐diabetic comparator group, pooled tobacco use prevalence was 24.2% among non‐T2D adults and 21.5% among T2D adults [5]. People with T2D were only modestly less likely to use tobacco (pooled OR 0.74, 95% CI 0.61–0.88), and in South Asia the direction was reversed, with T2D patients slightly more likely to smoke than the general population [5]. In other words, a diagnosis of diabetes has not translated into meaningfully lower rates of tobacco use. For a condition characterized by heightened cardiometabolic vulnerability, this level of smoking exposure translates into disproportionate downstream harm, amplifying risks of vascular complications, disability, and premature death.

Several caveats warrant consideration. Most T2D‐specific prevalence estimates derive from higher‐income countries. Data from many low‐ and middle‐income countries remain limited, particularly in Sub‐Saharan Africa and parts of Latin America. In the Roderick et al. review, only three studies were available from Latin America and none from low‐income countries [5]. Definitions of tobacco use, survey methodologies, and study periods varied across included studies. In addition, the African estimate comes from a separate review with different inclusion criteria [4].

Despite these limitations, the overall pattern is consistent: tobacco use among adults with T2D remains common across regions and is not meaningfully lower than in the general population.

The distribution of this burden is likely to shift further. Diabetes prevalence is rising most rapidly in low‐ and middle‐income countries [1], which already account for the majority of global tobacco use. In settings where cessation services, pharmacotherapy access, and continuity of care are limited, co‐exposure to metabolic risk and tobacco smoke toxicants becomes increasingly concentrated. The consequence is widening inequity, with preventable vascular events and premature mortality disproportionately affecting populations least equipped to mitigate them.

## 3 The Clinical Case: Smoking as a Driver of Diabetes Complications

In T2D, smoking is not a peripheral lifestyle factor. It is a clinically significant risk amplifier. Smoking not only increases the risk of developing T2D but also worsens outcomes after diagnosis. Among patients with diabetes, smoking is associated with higher risks of cardiovascular events, peripheral arterial disease, and mortality [6]. Meta‐analytic evidence shows that, among people with diabetes, active smoking is associated with markedly higher risks of all‐cause and cardiovascular mortality, as well as increased rates of cardiovascular events, compared with non‐smoking [7]. These associations are particularly consequential because they operate on an already elevated baseline risk. When the underlying probability of myocardial infarction or stroke is high, even modest relative increases translate into large absolute numbers of excess events and preventable deaths.

Smoking cessation is therefore a clinical priority. Evidence synthesis shows that former smokers with diabetes have meaningfully lower risks of mortality and cardiovascular events than current smokers [7]. A scoping review of the broader evidence further confirms that quitting smoking is associated with improvements across multiple diabetes‐related complications, including microvascular and macrovascular endpoints [8]. Cessation is not merely theoretically beneficial; it is associated with measurable reductions in the outcomes that drive morbidity and mortality in this population [9]. Beyond vascular outcomes, smoking also alters the metabolism of several antidiabetic medications, with potential implications for drug efficacy; cessation may therefore improve pharmacological diabetes management independently of its cardiovascular benefits [10]. These benefits warrant positioning cessation as a core component of diabetes risk management rather than as an optional lifestyle recommendation. Yet, in practice, cessation support is often deprioritized. Competing clinical demands, limited time, and assumptions about low success rates may contribute to therapeutic inertia. In a population at high cardiovascular risk, such inertia is difficult to justify and may perpetuate avoidable harm.

## 4 Why Smoking Cessation Remains Undertreated in Diabetes Care

**FIGURE 2:**
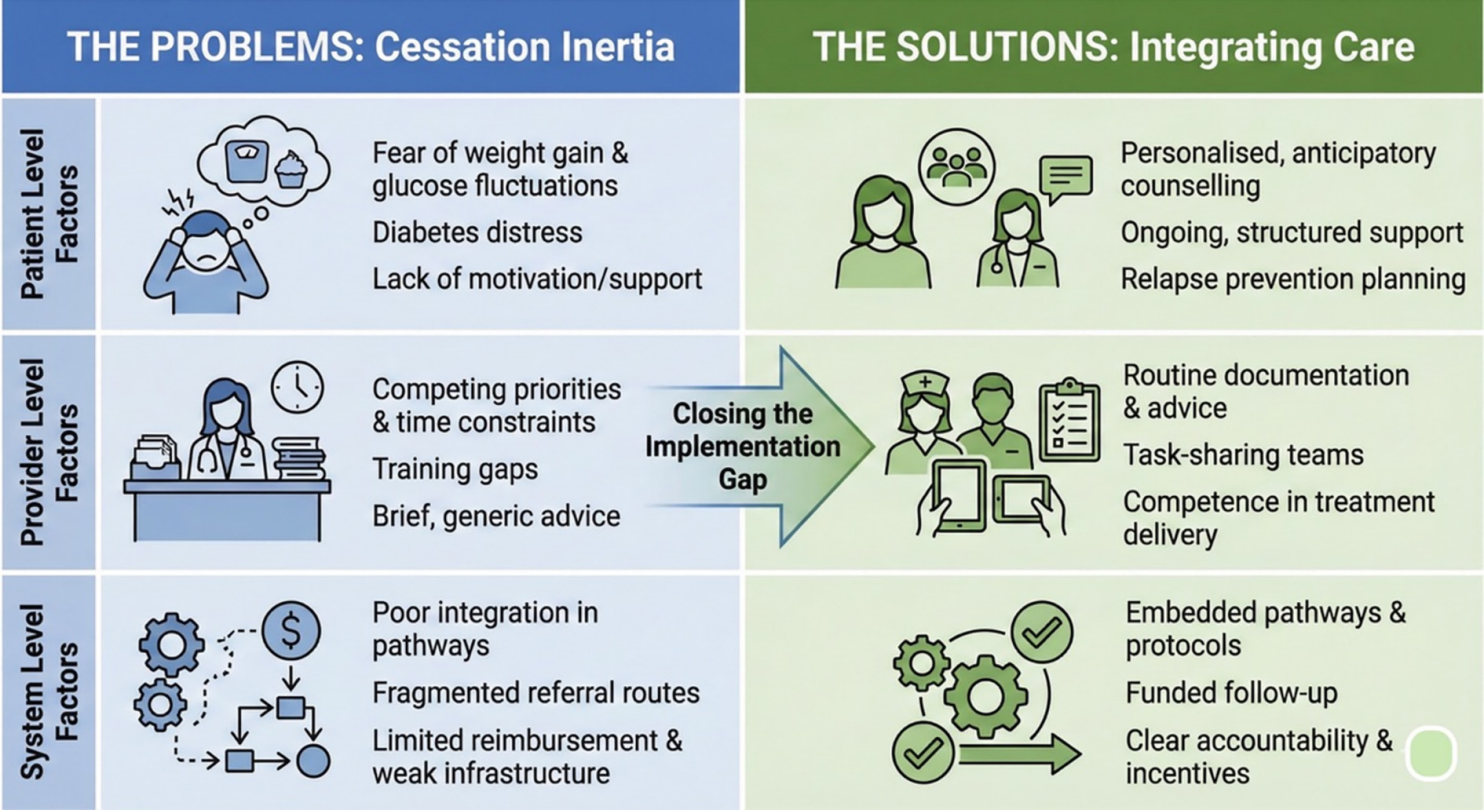
A Multilevel Framework for Integrating Smoking Cessation into Routine Diabetes Care.

If the clinical rationale is clear, why does smoking cessation remain undertreated in diabetes care? The barrier lies less in evidence gaps and more in failures of implementation (Figure 2).

At the patient level, barriers are well recognized. Many individuals living with T2D fear post‐cessation weight gain and short‐term deterioration in glycemic control [11]. These concerns are clinically plausible. Smoking cessation is commonly accompanied by increased appetite, weight gain, and transient metabolic fluctuations, particularly in the early weeks. Without anticipatory counseling and structured follow‐up, these changes can undermine motivation and precipitate relapse. In addition, diabetes‐related distress, anxiety, and depression may reinforce smoking as a coping strategy, deepening nicotine dependence and reducing the likelihood of successful unassisted cessation [3].

At the provider level, competing clinical priorities and training gaps persist. Diabetes care teams are well positioned to support repeated quit attempts, yet smoking cessation is rarely delivered in a systematic or sustained manner. In busy clinics, support often takes the form of brief admonitions, generic referrals, or deferred discussions that never translate into treatment engagement. Such approaches are predictably ineffective. Nicotine dependence is a chronic, relapsing condition that requires repeated intervention, structured behavioral support, pharmacotherapy where appropriate, and continuity of care. Without these elements, smoking cessation support remains episodic rather than therapeutic.

At the system level, smoking cessation remains poorly integrated within diabetes care pathways. Reimbursement is frequently limited, referral routes are fragmented, and follow‐up infrastructure is weak or inconsistently implemented. Without clinical prompts, standardized protocols, and accountability mechanisms, smoking status goes undocumented, support is delayed, and relapse is overlooked. The consequence is a treatment gap embedded within care delivery systems rather than within individual clinical encounters.

## 5 Towards Integration: Operationalizing Cessation Within Diabetes Care

Addressing this gap does not require a single universal model. Rather, it requires the application of established diabetes care logic: systematic identification, personalized advice, readiness‐based planning, prompt initiation of evidence‐based support, and scheduled follow‐up that anticipates relapse.

Tobacco smoking should be treated as a core diabetes care target, documented and addressed alongside HbA1c, blood pressure, LDL cholesterol, and weight. At a minimum, smoking status should be recorded at every diabetes review, accompanied by clear and personalized cessation advice. Patients ready to quit should be offered structured behavioral and pharmacological support rather than generic encouragement. Those not yet ready should still receive follow‐up plans that maintain engagement and periodically reassess readiness. Early follow‐up during the high‐risk cessation window, when withdrawal symptoms and relapse risk are greatest, is particularly important.

Workforce development is essential. Diabetes care teams require the competence, confidence, and clinical mandate to deliver cessation support directly, rather than relying solely on external referral pathways. Task‐sharing models can improve scalability and continuity: diabetes educators and nurses can deliver brief interventions, behavioral support, and follow‐up, while physicians focus on risk stratification, pharmacotherapy, and treatment escalation. Health systems must resource and embed cessation pathways within diabetes services. Without dedicated funding, reimbursement structures, and service integration, even well‐established clinical guidance remains aspirational rather than operational.

## 6 The Role of Harm Reduction When Cessation Is Not Achieved

Complete cessation of combustible tobacco should remain the primary goal in T2D management. However, care pathways must also account for patients who are unwilling or unable to quit despite repeated, supported attempts, or who lack access to first‐line cessation treatment [12]. In such situations, clinicians face a risk‐management decision rather than an ideal therapeutic choice. Established pharmacotherapies, including nicotine replacement therapy, bupropion, and varenicline, remain the cornerstone of cessation support and may improve quit rates compared with unaided attempts [13]. Nonetheless, long‐term abstinence remains difficult to sustain for many individuals, and relapse is common.

Within this context, alternative nicotine delivery systems have been evaluated as smoking cessation aids [14, 15]. Evidence syntheses indicate that nicotine e‐cigarettes can increase smoking cessation rates compared with nicotine replacement therapy in some clinical trials [14]. Indeed, cross‐sectional data suggest that e‐cigarette use is already prevalent among people with diabetes, with differential patterns of association compared with combustible smoking [16, 17, 18]. Where these products are used, the clinical objective should be explicit: to support a complete transition away from combustible tobacco, discourage sustained dual use, and reassess readiness for full cessation at each clinical review.

## 7 Conclusion

Smoking among people living with T2D is a major, modifiable driver of cardiovascular morbidity and premature mortality. Yet, it remains insufficiently prioritized within routine diabetes care, largely because cessation has not been operationalized as a core treatment outcome. Positioning tobacco use alongside glycemic control, blood pressure, and lipid management, and systematically supporting cessation and relapse prevention, offers an immediate opportunity to reduce preventable complications. Where repeated quit attempts are unsuccessful, pragmatic harm‐reduction strategies may be considered, provided the primary goal remains complete transition away from combustible tobacco. As diabetes prevalence rises most rapidly in settings with limited cessation infrastructure, closing this implementation gap is not only clinically sound but essential to equitable chronic disease management.

## Author Contributions

All authors meet the authorship criteria of the International Committee of Medical Journal Editors (ICMJE) and have approved the final manuscript. Yusuff Adebayo Adebisi conceptualized the paper, conducted the literature synthesis, and drafted the manuscript. Anoop Misra contributed clinical expertise on diabetes care and critically revised the manuscript. Riccardo Polosa conceptualized the paper, contributed expertise on smoking cessation and harm reduction and critically revised the manuscript. All authors agree to be accountable for all aspects of the work.

## Funding

The authors have nothing to report.

## Ethics Statement

The authors have nothing to report.

## Conflicts of Interest

Anoop Misra is the editor‐in‐chief of Diabetes & Metabolic Syndrome: Clinical Research & Reviews (Elsevier) and declares no conflicts of interest. Yusuff Adebayo Adebisi has previously received funding through the Tobacco Harm Reduction Scholarship and the Kevin Molloy Fellowship, both awarded by Knowledge‐Action‐Change (KAC), an independent public health organization based in the United Kingdom that is funded by Global Action to End Smoking, an independent US non‐profit 501(c)(3) grant‐making organization. Riccardo Polosa has received grants from multiple public, private, and non‐profit sources, including U‐BIOPRED, AIR‐PROM, IRIS, the Foundation for a Global Action to End Smoking, Pfizer, GlaxoSmithKline, CV Therapeutics, NeuroSearch A/S, Sandoz, Merck Sharp & Dohme, Boehringer Ingelheim, Novartis, Arbi Group Srl., Duska Therapeutics, Forest Laboratories, and ministerial grants funded by NextGenerationEU of the European Union; has received consultancy fees from Pfizer, Boehringer Ingelheim, Duska Therapeutics, Forest Laboratories, CV Therapeutics, Sermo Inc., GRG Health, Clarivate Analytics, Guidepoint Expert Network, and GLG Group; receives textbook royalties from Elsevier; is involved in a patent application for ECLAT Srl.; is the founder of the Center for Tobacco Prevention and Treatment and the Center of Excellence for the Acceleration of Harm Reduction at the University of Catania; serves as a pro bono scientific advisor for Lega Italiana Anti Fumo and the International Network of Nicotine Consumers Organizations; is chair of the European Technical Committee for Standardization on requirements and test methods for emissions of electronic cigarettes (CEN/TC 437, WG4); and is a scientific advisor of the non‐profit Foundation RIDE2Med.

## Data Availability

Data sharing not applicable to this article as no datasets were generated or analyzed during the current study.
